# Detection and Concentration of Neonicotinoids and Other Pesticides in Honey from Honey Bee Colonies Located in Regions That Differ in Agricultural Practices: Implications for Human and Bee Health

**DOI:** 10.3390/ijerph19138199

**Published:** 2022-07-05

**Authors:** Gilda Ponce-Vejar, S. Lizette Ramos de Robles, José Octavio Macias-Macias, Tatiana Petukhova, Ernesto Guzman-Novoa

**Affiliations:** 1 Departamento de Ciencias Ambientales, Centro Universitario de Ciencias Biológicas y Agropecuarias, Universidad de Guadalajara, Guadalajara 44600, Mexico; gildapov@gmail.com; 2 Centro de Investigaciones en Abejas (CIABE), Centro Universitario del Sur, Universidad de Guadalajara, Ciudad Guzmán 49000, Mexico; joseoc@cusur.udg.mx (J.O.M.-M.); eguzman@uoguelph.ca (E.G.-N.); 3 Department of Population Medicine, University of Guelph, Guelph, ON N1G 2W1, Canada; tpetukho@uoguelph.ca; 4 School of Environmental Sciences, University of Guelph, Guelph, ON N1G 2W1, Canada

**Keywords:** neonicotinoids, pesticides, honey, honey bee health, human health, environmental pollution, Mexico

## Abstract

This is a preliminary study conducted to analyze the presence and concentration of pesticides in honey obtained from honey bee colonies located in two regions with managed ecosystems that differ in the intensity and technification of agricultural practices. Fourteen pesticides at variable concentrations were detected in 63% of the samples analyzed. The pesticides most frequently found at higher concentrations were insecticides (neonicotinoids, followed by organophosphates), herbicides, and fungicides. The number, frequency, and concentration of pesticides were higher in samples collected from hives located where intensive and highly-technified agriculture is practiced. Forty-three percent of the samples from that zone had residues of imidacloprid, compared with only 13% of the samples from the less-technified zone. Furthermore, 87.5% of those samples had imidacloprid concentrations that were above sublethal doses for honey bees (>0.25 ng/g) but that are not considered hazardous to human health by the European Commission. The results of this study suggest that honey can be used as a bioindicator of environmental contamination by pesticides, which highlights the need to continue monitoring contaminants in this product to determine the risks of pesticide impacts on pollinator health, on ecosystems, and on their potential implications to human health and other non-target organisms.

## 1. Introduction

The world population has increased from 2.6 billion in 1950 to more than 7.7 billion in 2019 and is forecasted to reach 9.7 billion by 2050 [1]. Parallel to this population growth, the demand and production of agricultural crops has increased. However, increasing the production of agricultural products is a challenging task that has led to the loss of biodiversity due to the transformation of natural ecosystems into managed ones and to the intensification of agricultural practices [2]. Agricultural production systems vary in their degree of technification. There are traditional, low-technified agrosystems, which rely solely on natural rainfall and minimize the use of pesticides and other agrochemicals [3]. Conversely, there are highly-technified agrosystems that protect crops with covers of translucent synthetic materials (e.g., greenhouses, macro-tunnels, etc.), or control the temperature of crops (e.g., avocado orchards), and that heavily rely on the use of agrochemicals [4]. Nevertheless, whatever the type of agricultural system, crops can be affected by diseases and pests, so it is necessary to resort to the use of pesticides [5] to reduce or avoid the loss of crops. However, the use of pesticides has generated problems ranging from toxicity to humans and wildlife and to the alteration of ecosystems [6]. In addition, although pesticides were designed to kill organisms harmful to crops, they also harm beneficial insects such as bees.

A new family of pesticides, the so-called neonicotinoids, was incorporated to the market in the 1990s. Neonicotinoids are persistent and systemic insecticides that, with lower doses than those of other pesticides, protect crops from harmful insects [7]. Neonicotinoids have rapidly replaced the use of organophosphates, carbamates, pyrethrins, and pyrethroids [8], becoming the most widely-used insecticide worldwide. However, there is evidence indicating that neonicotinoids represent a threat to human health [9], to beneficial insects (including bees), and to the environment [10,11].

Recent studies have evidenced risks of neonicotinoid toxic effects to human health, including neurotoxicity, hepatotoxicity, immunotoxicity, genotoxicity, and reproductive system impairments [12], as well as neurodevelopmental immunotoxicity and inflammation of the central nervous system [13]. However, despite the above evidence, epidemiological studies on the effects of neonicotinoids in humans are still very limited.

Regarding the impact of neonicotinoids on pollinators, massive losses of honey bee colonies have been documented since neonicotinoids began to be used in agricultural settings [14,15,16,17]. Although there are several factors that affect the survival of bees, the use of neonicotinoids is one of the factors most frequently associated with the death of these insects [18,19] because they are highly toxic and persistent, even at very low concentrations [8,11,20]. Some neonicotinoids are capable of causing sublethal effects in bees, such as reduced lifespan, immune responses, learning, and foraging, as well as increased susceptibility to viruses [21,22,23,24,25,26,27]. These effects may impair the bees’ ability to collect resources and pollinate wild plants and commercial crops, which may have a negative impact on managed and natural ecosystems [28].

The foraging behavior of bees allows them to cover large areas of land, which turns them into sentinels of the environment [29,30], although this behavior puts bees in a vulnerable situation, as they are often exposed to pesticides [31,32]. Several studies have used honey as an indicator of the environmental contamination of heavy metals [33], ionic compounds [34], radioactive compounds [35], and pesticides [36] in a number of countries. Because of their foraging behavior, honey bees are exposed to neonicotinoids and other pesticides constantly, all over the world [37].

Mexico is an important honey producer; however, only one study has been previously conducted in this country to determine the presence of pesticides in hive products [38]. The study was conducted in three regions of Mexico that did not include the western area of the nation, which is the main industrialized agriculture area of Mexico. Additionally, the study used detection levels considered relatively high for sublethal effects on bees.

This study was conducted to analyze the presence and concentration of neonicotinoids and other pesticides in honey samples collected from hives located in two regions of western Mexico that differ in the technification of agricultural systems, as well as to determine their potential risk to human and honey bee health.

## 2. Materials and Methods

### 2.1. Study Areas

This study was carried out in the state of Jalisco, Mexico (18°58′ N, 105°43′ W). Jalisco is Mexico’s most productive agricultural state, in which low-technified and highly-technified agriculture are practiced. Highly-technified agricultural systems have developed rapidly and today account for the production of more than 60% of agricultural foodstuff grown under this modality, mainly in municipalities located in the southern part of the state [39,40]. Therefore, for the purposes of this study, the state was divided into two regions according to the type of agriculture practiced: the north zone, where agricultural production is seasonal and low-technified, and the south zone, where intensive technified agriculture is practiced in greenhouses, tunnels, and orchards [38,39].

### 2.2. Sample Collection

**Figure 1:**
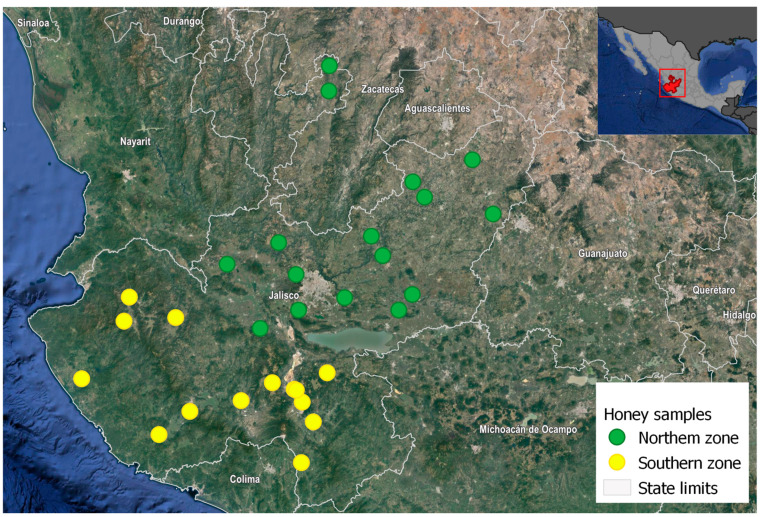
Study area. Honey samples were collected from 30 different hives located in the north (low technified agriculture) and south (highly technified agriculture) zones of Jalisco, Mexico. Source: Map modified from Google Earth [42].

Honey samples were collected from 30 hives (16 in the north zone and 14 in the south zone). The criterion for choosing the sampling places was based on selecting the municipalities that contributed >60% of the honey produced in 2016 [41], which are distributed in 12 regions along the state (Figure 1).

Fixed apiaries from two to four municipalities were selected in each region, and the samples were collected between April and May 2018. From each selected apiary, a honey sample was collected from a randomly chosen colony. The sample was collected from the hive storage area (supers), where a comb section containing capped honey (10 cm × 10 cm) was cut out with a food-grade plastic knife. The sample was placed in a sealable plastic bag (Ziploc^®^), and geo-referencing data from the apiary were recorded. The samples were kept in a cooler with ice, transported to the laboratory, and transferred to 30-mL glass vials that were previously sanitized with 96° ethanol and dried in an oven (model FE-131, FELISA, Zapopan, Mexico). For pesticide analysis, samples were shipped to the University of Guelph’s Food and Agriculture Laboratory in Ontario, Canada.

### 2.3. Laboratory Analysis of Honey Samples

The samples were analyzed at the University of Guelph’s Agriculture and Food Laboratory (AFL), which is part of the Canadian Association of Laboratory Accreditation (CALA) and the International Organization of Standardization and International Electrotechnical Commission (ISO/IEC 17025). The AFL is accredited by the Standards Council of Canada (SCC).

#### 2.3.1. Identification Criteria, Quality Control, and Validation of the Analytical Procedure

The samples were analyzed by Liquid Chromatography/Electrospray Ionization-Tandem Mass Spectrometry (LC/ESI-MS/MS), which is a useful method for the determination of pesticides in foods according to Wang and Leung [43]. The protocol used determines the presence of 236 pesticides, including fipronil and the neonicotinoids acetamiprid, clothianidin, imidacloprid, dinotefuran, thiacloprid, and thiamethoxam. Pesticides were extracted from the honey samples using the quick, easy, cheap, effective, rugged, and safe (QuEChERS) extraction method.

A representative sample was extracted into 1% acetic acid (CH_3_-COOH in acetonitrile in the presence of anhydrous sodium acetate, C_2_H_3_NaO_2_) and magnesium sulfate (MgSO_4_). Cleanup was performed on the supernatant using dispersive solid-phase extraction (dispersive-SPE) with MgSO_4_ and primary and secondary amine exchange material (PSA). The concentrated extract was quantified by LC-MS/MS using matrix-matched standard curves and isotopically-labeled internal standards.

#### 2.3.2. Performance Results of the Analysis Methodology

Quantification was based on standard calibration curves with the use of an isotopically-labeled standard or a chemical analogue as the internal standard for method accuracy. Performance parameters, including overall recovery, intermediate precision, and measurement uncertainty, were evaluated on the basis of a nested design. The performance results were calculated by using a compiled SAS program that provides a procedure for handling a large number of calculations. The method provides an analytical range of 1–100 mg/kg with the lowest concentration level at 1 mg/kg for all pesticides, except for aclonifen at 5 mg/kg [43].

The pesticides found were grouped into families according to the databases of the Pesticide Action Network [44], and their frequency and concentrations (ng/g) were analyzed.

### 2.4. Statistical Analyses

Descriptive parameters were obtained for the identified and quantified pesticides. The frequency of the different families of pesticides identified was compared using χ^2^ tests to determine significant associations between the different families of pesticides and between the two agricultural zones studied. To analyze the concentration data of the different pesticides found in the two zones, the assumption of normality was verified with the Shapiro-Wilk test, and the assumption of homogeneity was verified with the Bartlett test. Both assumptions were unsatisfactory. Therefore, the concentrations of the pesticides were compared with the Wilcoxon non-parametric test. All data were analyzed with R version 3.3.1 with a significance level of <0.05. Additionally, the concentrations of the pesticides detected were compared with the Maximum Residue Limits (MRLs) established by the European Commission (EC), as a regulation towards the care and vigilance of residual traces of pesticides that can be found and tolerated in food for human consumption [45]. In addition, the concentrations of the pesticides detected were compared with those of studies that have shown damage to the health and behavior of bees at sublethal doses.

## 3. Results

### 3.1. Pesticides Detected

**Figure 2:**
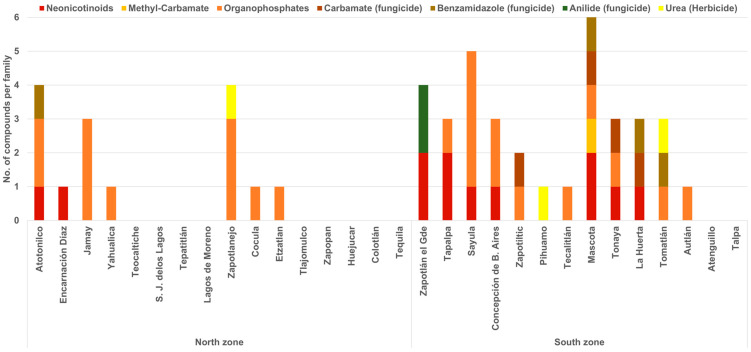
Number of compounds of each of seven families of pesticides detected in honey samples collected from honey bee hives in 16 municipalities of the north zone and in 14 municipalities of the south zone in Jalisco, Mexico.

Pesticides were detected in 63% of the honey samples, and 14 different compounds were identified, 74% of which were insecticides, 20% fungicides, and 6% herbicides. Acetamiprid and imidacloprid were the only two neonicotinoid insecticides detected in addition to organophosphates and methyl-carbamate. Imidacloprid and coumaphos were the pesticides most frequently found (27%), whereas the least frequent were formetanate, monocrotophos, acephate, boscalid, and fenhexamid (3–7%). The pesticides found in the municipalities of the two zones studied are shown in Figure 2.

### 3.2. Frequency of Pesticides Detected by Zone

**Figure 3:**
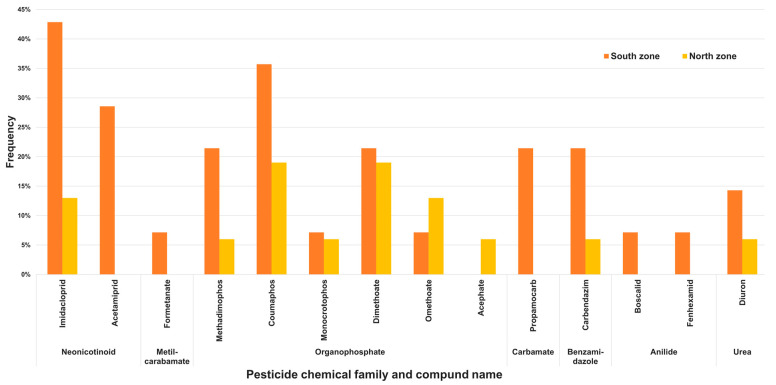
Frequency of pesticides detected in honey samples collected from honey bee hives from the north and south zones of Jalisco, Mexico.

There were significant differences for pesticide frequency between the two zones (χ^2^ = 17.1, *p* < 0.05). Pesticides were detected in 86% of the south zone samples, compared to only 44% in the north zone samples. Moreover, neonicotinoids were detected more frequently in the south zone than in the north zone. For example, imidacloprid was found in 43% of the south zone samples, compared to only 13% of the north zone samples. The most frequent compounds in samples from the north zone were dimethoate and coumaphos (19%), and the least frequent were monocrotophos, acephate, carbendazim, and diuron (6%). In the south zone, the pesticide most frequently detected was imidacloprid (43%), whereas the pesticides least frequently detected were formetanate, boscalid, and fenhexamid (3.3%). Imidacloprid was detected significantly more frequently than these three pesticides (χ^2^ = 4.7, *p* < 0.05; Figure 3).

### 3.3. Pesticide Concentrations

The concentrations of the 14 pesticides detected in honey samples were variable. The highest concentrations measured were for formetanate and acetamiprid, whereas the lowest corresponded to omethoate and propamocarb (Table 1).

These concentrations also varied by zone. In the north zone, imidacloprid and dimethoate had the highest concentrations, while in the south zone the highest concentrations were for formetanate and acetamiprid, although the differences were not significant (*p* > 0.05). In general, the pesticides had higher concentrations in the south zone than in the north zone (up to 5 times higher). For example, the concentrations for imidacloprid and coumaphos in the south zone were more than three times higher than in the north zone.

### 3.4. Pesticide Levels and Risk to Human Health

**Figure 4:**
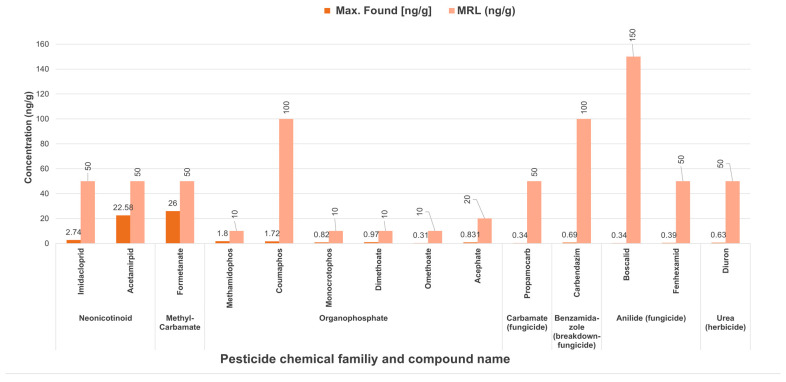
Concentrations of pesticides in samples of honey (ng/g) from Jalisco, Mexico, compared with MRL established by the European Commission [45].

The concentrations of the pesticides detected in the honey samples were compared with the MRL established by the EC, and it was found that, in average, those concentrations were 58 times lower than the MRL (Figure 4).

### 3.5. Pesticide Levels and Risk to Honey Bee Health

**Figure 5:**
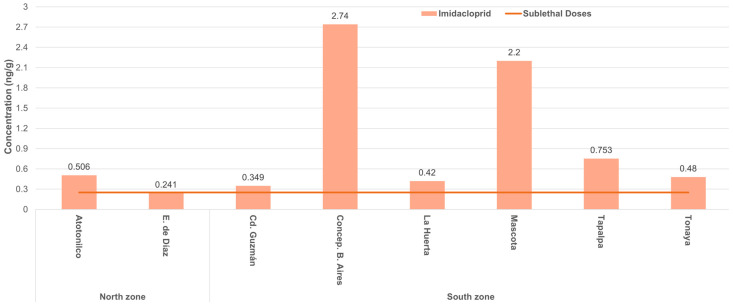
Concentrations of imidacloprid in honey samples collected from honey bee hives from the north and south zones of Jalisco, Mexico, compared with sublethal doses of the pesticide for honey bees (red line), according to Williamson and Wright (2013) [46].

For honey bees, 87.5% of the honey samples where imidacloprid was detected, and one sample where coumaphos was detected, had levels of the pesticides that were above sublethal doses demonstrated by Williamson and Wright [46] (Figure 5).

## 4. Discussion

The results of this study show that pesticide residues, particularly those of neonicotinoids, can be present in different regions and in greater frequency and concentration in areas where more technology is applied and more pesticides are used in agricultural production systems, such as in southern Jalisco, Mexico. This finding is particularly novel for that region of Mexico and should be a warning sign of the potential risk that pesticides represent in terms of negative effects on the environment, human health, and bee health [9,10,11,47].

More pesticides and in higher concentrations were found in honey samples from the south zone than in samples from the north zone. These results are likely related to the type of agriculture practiced in them. In the north zone, growers practice low-technified agriculture, and pesticide use is less intensive, while in the south zone, where intensive agricultural production systems are implemented, the use of pesticides is high and continuous [40]. The proper use of pesticides should not pose significant threats or major detrimental effects to the environment and fauna. However, the misuse of pesticides, their mixtures, or their application for unjustified preventive purposes [10], that is, the application of the products even without evidence of pest damage in a crop, might cause synergistic toxic effects to various organisms [48]. The fact that compounds such as imidacloprid and coumaphos had been found more frequently than other compounds in honey samples could imply a potential risk for fauna in general, due to their high persistence in the environment [20]. Due to their persistence in the environment, neonicotinoids have been detected in similar monitoring carried out in different world regions, where there are different strategies in regulatory frameworks [30,49]. A study of pesticide residues in honey bees, bee bread, and beeswax from French hives indicated the high contribution of field pesticides (farmer applied) to bee exposure within the hive itself. The comparison of the contamination before and after the restriction of neonicotinoid use in France showed a decrease in the frequency of detection of these molecules, mainly at low levels [49]. In another study, Traynor et al. [30] assessed the pesticide pollution of pollen collected by honey bees throughout the USA, determining that bees were exposed to 120 different pesticide products, including neonicotinoids.

Imidacloprid, in particular, is one of the most commonly used neonicotinoid insecticides and, thus, has been found in honey samples in previous reports [8,36]. A recent example is the study of Scripcâ and Amariei [50], which showed that neonicotinoids were the most commonly detected pesticides in monofloral and multifloral honey samples and that the main source of these pesticides were agricultural crops. Similarly, Valdovinos-Flores et al. [38] also found imidacloprid in honey samples collected in central Mexico, where pesticides are widely used in agricultural settings.

The results of this study suggest that honey may be a good bioindicator of environmental contamination of a wide area surrounding honey bee hives [29,32]. When foraging, bees can cover an area of between 914 and 3935 hectares, considering that, on average, they forage at distances between 1.6 and 3.2 km from their hive [51,52]. Other studies have also shown that honey can be used to obtain parameters of environmental pollution [34,35,36,40], and it can even be used as a bioindicator of heavy metals and radionuclides [53]. The results of this work showed that some locations where honey samples were collected can still be found apparently free of contamination by pesticides, since in nine of the samples from the north zone no polluting residues were detected, while in the south zone, the majority of the honey samples contained residues of at least one chemical compound, except for two samples that were collected from natural environments. These results are likely due to the fact that less intensive and technified agriculture is practiced in the north zone compared to the south zone, which results in pesticide-free geographic regions, at least temporarily, and under the detection thresholds of the analytical methods used in this study.

The concentrations of the pesticides detected in the honey samples from this study were below the MRLs authorized for food products of human consumption [45]. However, six of the fourteen pesticides detected (imidacloprid, methamidophos, monocrotophos, dimethoate, omethoate, and acephate) are classified as highly hazardous because of their acute or chronic toxicity to human health, according to criteria established by WHO, IARC, and EPA [54]. Despite the fact that honey from the regions studied can be considered as “suitable” for human consumption, given that the concentrations of these pesticides did not exceed MRLs, we cannot claim “zero” risk, because the contamination of pesticides is evident in 63% of the samples. In other words, an absence of risk cannot be claimed, which is why it may be necessary to consider the accumulated risk as a result of frequent intake of pesticides in honey or in other agri-foods that are consumed in the studied regions.

The neonicotinoid imidacloprid has not yet been included in the toxicity classification lists elaborated by the aforementioned organizations. Therefore, for the purposes of this study, we used the MRLs established by the EC. However, based on the results of this research and on those of other reports where its potential toxicity has been documented [12], as well as based on its potential carcinogenic effects [55], its classification in those lists seems necessary. In this study, acetamiprid and imidacloprid were the most frequent neonicotinoids in honey samples and were found more frequently in the south zone, in combination with 12 other pesticides, although none of these exceeded MRLs. However, this study was restricted to analyze the presence and levels of neonicotinoids and other pesticides only in honey. Therefore, it did not estimate risks due to the daily intake of the insecticides in other foods produced in the same regions. Nevertheless, if further research confirms damage to human health with low doses and the exposure of the studied pesticides, the MRLs established so far could be decreased. That would imply that the concentrations found in this study could potentially represent a risk to human health.

Concentrations of some of the pesticides detected in honey in this study could represent a risk for bees, particularly those found for imidacloprid. Yang et al. [56] reported that the exposure of bee larvae to 0.04 ng of imidacloprid had long-term detrimental consequences in the memory of adult bees. This amount of imidacloprid is at least four times lower than the level the larvae of colonies from the south zone would have been exposed to when consuming honey for their development [57], considering the concentrations of neonicotinoid found in the honey of those colonies. That amount of imidacloprid would also be similar to the levels bees from colonies of the north zone would have been exposed to. In another study, Williamson and Wright [46] found that imidacloprid at concentrations of 0.25 ng/g of food caused negative effects on learning in honey bees. In this study, the mean concentration of imidacloprid in honey from colonies of the south zone was almost five times higher than 0.25 ng/g. In other studies, intakes of imidacloprid by adult bees, lower than those that bees of the colonies sampled in the south zone would have consumed from honey stored in honeycombs, resulted in a reduction in foraging behavior and in communication dances used by field bees to recruit nestmates to foraging sites [58,59]. In addition, Morfin et al. [21,22] tested clothianidin at a concentration and consumption similar to that the bees in the south zone would have been exposed to for imidacloprid, and they found a reduction in hygienic and grooming behavior in the exposed bees. These behaviors contribute to restraining the population growth of the parasitic mite *Varroa destructor*, which is the most damaging biotic factor of honey bee health worldwide. Therefore, it can be inferred that the concentrations of imidacloprid found in honey in this study, particularly in colonies from the south zone, represent a risk to the health and behavior of honey bees.

Acetamiprid, the other neonicotinoid pesticide detected in honey samples, had lower concentrations than those that have been shown to cause harmful effects on bee health. However, it must be considered that more than one pesticide was found in 88% of the samples, which could represent a risk of synergistic effects due to the combined action of multiple pesticides. Coumaphos, for example, is a highly toxic pesticide that, in combination with other cholinergic pesticides, may impair olfactory learning and memory in honey bees [46]. One of the honey samples in this study had concentrations twice higher than those tested by Williamson and Wright [46]. Of the fourteen pesticides detected, six (imidacloprid, methamidophos, monocrotophos, dimethoate, omethoate, and acephate) are classified by the EPA as hazardous because they are highly toxic to bees [54]. This should be an alert for beekeepers and farmers about the inappropriate use of pesticides. Exposure to hazardous pesticides even at low concentrations (nanoconcentrations) can be an additional stressor that may affect the productivity and health of honey bee colonies, particularly for those located in regions of intensive and highly technified agriculture, such as the south zone in this study. This can also represent a risk for the maintenance of agri-food systems and biodiversity [28].

In this study, 14 pesticides detected in honey varied in frequency and concentration between two zones with different levels of agricultural technification. These results evidence the contamination of the environment with toxic products that may represent a potential risk to the health of honey bees and potentially to human health. These results also show that honey can be used as a bioindicator of environmental contamination with pesticides in different regions. Additionally, this information could be useful in the preparation of risk analyses and in the development of policies aimed at regulating and controlling the use of pesticides in agricultural production, particularly in regions with intensive, technified agriculture.

## 5. Conclusions

This is a preliminary study that analyzed the presence and concentration of pesticides in honey collected from honey bee colonies located near managed ecosystems that differ in the intensity and technification of agricultural practices. Fourteen pesticides at variable concentrations were detected in 63% of honey samples analyzed. The pesticides most frequently found and at higher concentrations were neonicotinoids, followed by organophosphates, herbicides, and fungicides, all of which can affect human health and bee health and can contaminate the environment. The results of this study are evidence of the presence of highly toxic insecticides such as imidacloprid, with higher frequency and concentration in the south zone, where intensive and highly-technified agriculture is practiced. Honey can be used as an indicator of environmental contamination to determine the risks of pesticide impacts on pollinator health, on ecosystems, and on their possible implications for human health and other non-target organisms. Although preliminary, this study could serve as a baseline for more comprehensive and larger studies aimed at determining the risk that pesticides pose to the environment and food production. Future studies should be longitudinal with the periodical collection and analysis of samples all-year long, which would provide information on the risks of pesticide contamination during different seasons of the year.

## Figures and Tables

**Table 1:** Pesticides by chemical family detected in the north and south zones of Jalisco, Mexico, and mean concentration of each pesticide in ng/g of honey ± SE.

Jalisco’s Zone	Compound	Chemical Family	Use	Number of Samples with Pesticide	Number of Samples without Pesticide	Mean Concentration ± SE ^a^
North	imidacloprid	neonicotinoid	Insecticide	2	14	0.37 ± 0.13
	metadimophos	organophosphate	Insecticide	1	15	0.2 ± 0
	coumaphos	organophosphate	Insecticide	3	13	0.15 ± 0.06
	monocrotophos	organophosphate	Insecticide	1	15	0.26 ± 0
	dimethoate	organophosphate	Insecticide	3	13	0.32 ± 0.09
	omethoate	organophosphate	Insecticide	2	14	0.24 ± 0.07
	acephate	organophosphate	Insecticide	1	15	0.83 ± 0
	carbendazim	benzimidazole	Fungicide	1	15	0.12 ± 0
	diuron	urea	Herbicide	1	15	0.19 ± 0
South	imidacloprid	neonicotinoid	Insecticide	6	8	1.16 ± 0.42
	acetamiprid	neonicotinoid	Insecticide	4	10	7.55 ± 5.19
	formetanate	methyl-carbamate	Insecticide	1	13	26 ± 0
	metadimophos	organophosphate	Insecticide	3	11	1.02 ± 0.42
	coumaphos	organophosphate	Insecticide	5	9	0.52 ± 0.30
	omethoate	organophosphate	Insecticide	1	13	0.12 ± 0
	propamocarb	carbamate	Fungicide	3	11	0.26 ± 0.04
	carbendazim	benzimidazole	Fungicide	3	11	0.36 ± 0.17
	boscalid	anilide	Fungicide	1	13	0.34 ± 0
	fenhexamid	anilide	Fungicide	1	13	0.39 ± 0
	diuron	urea	Herbicide	2	12	0.48 ± 0.16

(a) Mean concentration in ng/g ± standard error (SE) from positive samples for each compound.

## Data Availability

Data will be made available from the corresponding author upon reasonable request.
